# *In Vitro* Activity of 3 Commercial Bacteriophage Cocktails Against *Salmonella* and *Shigella* spp. Isolates of Human Origin

**DOI:** 10.20411/pai.v3i1.234

**Published:** 2018-05-29

**Authors:** Odette J. Bernasconi, Valentina Donà, Regula Tinguely, Andrea Endimiani

**Affiliations:** 1 Institute for Infectious Diseases, University of Bern, Bern, Switzerland; 2 Graduate School of Cellular and Biomedical Sciences, University of Bern, Bern, Switzerland

**Keywords:** commercial, bacteriophages, Salmonella, Shigella, cocktails

## Abstract

**Background:**

*Salmonella* and *Shigella* spp. are 2 of the most frequent and deadly enteric bacterial pathogens recorded worldwide. In developing countries *Salmonella* infections are responsible for many deaths annually and these mortality rates are prone to increase due to the emergence of resistance to antibiotics. In this overall scenario new alternative therapeutic approaches are needed.

**Methods:**

For the first time, we investigated the activity of 3 commercial bacteriophage cocktails (*INTESTI, Septaphage, PYO*) against a collection of contemporary *Salmonella* spp. (n = 30) and *Shigella* spp. (n = 20) strains isolated in Switzerland. Phage susceptibility was determined by implementing the spot test.

**Results:**

The overall susceptibility of *Salmonella* spp. to *INTESTI* and *Septaphage* was 87% and 77%, respectively. With regard to *Shigella* spp., the overall susceptibility to *INTESTI* and *Septaphage* was 95% and 55%, respectively. *PYO* was observed to be active against only 10% of *Salmonella* spp. but against 95% of *Shigella* spp.

**Conclusions:**

Our results seem promising, especially for the *INTESTI* biopreparation against *Salmonella enterica* infections. Nevertheless, such speculation should be supported by further *in vivo* studies to confirm efficacy and safety of the cocktails. We also emphasize the importance of large *in vitro* screening analyses aimed to assess the activity of such biopreparations against contemporary multidrug-resistant strains that are emerging worldwide.

## INTRODUCTION

*Salmonella* and *Shigella* spp. are the most frequently found and deadly enteric bacterial pathogens. For instance, each year 500,000 cases of diarrheal shigellosis and about 1.2 million cases of nontyphoidal salmonellosis with 380 deaths are recorded in the United States [1–4]. Moreover, in developing countries *Salmonella* infections are responsible for 1 million deaths annually and these mortality rates are likely to increase due to the emergence of resistance to commonly implemented antibiotics [5, 6]. In this overall scenario, new alternative and cost-effective therapeutic approaches are needed.

Bacteriophages are highly species-specific self-propagating viruses that can infect and lyse bacteria. Their employment is part of the standard medical practice in countries of the former Soviet Union, whereas in Western nations the use of phage therapy is unfamiliar, and this has led to a lack of studies analyzing efficacy and possible alternatives to antibiotics [7, 8].

Numerous *in vitro* and *in vivo* reports exploring both lytic activity and clinical effectiveness to control *Salmonella* infections are available. However, such analyses have exclusively used monophages and focused on reducing contamination of food stuffs or intestinal colonization in food animals [9–13]. With regard to *Shigella*, Mai *et al* tested a phage cocktail (ShigActive^TM^) in a mice model obtaining encouraging results [14].

To our knowledge, data regarding the *in vitro* activity of bacteriophage cocktails against large collections of *Salmonella* and *Shigella* spp. strains are still lacking. In this study, for the first time, we explored the *in vitro* activity of 3 commercially available bacteriophage cocktails currently implemented in the country of Georgia to treat human intestinal infections.

## METHODS

The following cocktails of sterile-filtrate phage lysates of different bacterial species were tested: *PYO Bacteriophage, INTESTI Bacteriophage* (Eliava Biopreparations, Tbilisi, Georgia; concentration of 10^5-6^ Plaque Forming Units, PFU/mL), and *Septaphage* (Biochimpharm, Tbilisi, Georgia; 10^5^ PFU/mL). *PYO* targets *Escherichia coli, Proteus* spp., *Pseudomonas aeruginosa, Staphylococcus* spp., and *Streptococcus* spp., whereas *INTESTI* and *Septaphage* target over 12 gastrointestinal pathogens, such as *Shigella, Salmonella, Proteus, Staphylococcus, Pseudomonas* spp. and different serovars of enteropathogenic *E. coli. PYO* is used to treat purulent skin and surgical, oral, enteral, and gynecological infections, whereas *INTESTI* and *Septaphage* are implemented for intestinal infections [15]. Notably, *INTESTI* is the only molecularly well-characterized phage cocktail [16].

The collection of strains tested during the present study included contemporary *Salmonella* (n = 30) and *Shigella* spp. (n = 20) isolated from human infections which occurred in Switzerland. Species identification (ID) was routinely obtained using the matrix-assisted laser desorption ionization-time of flight mass spectrometry (MALDI-TOF MS; Bruker). The ID confirmation and further typing were performed at the National Reference Laboratory for Enteropathogenic Bacteria and Listeria (Institute for Food Safety and Hygiene, Zurich, Switzerland). The antibiotic susceptibility profiles were obtained by disc-diffusion tests [17]. Most *Salmonella* spp. strains were pan-susceptible to tested antibiotics (ampicillin, ceftriaxone, cotrimoxazole, chloramphenicol, nalidixic acid, and ciprofloxacin), whereas only ceftriaxone was always active *in vitro* against isolates of *Shigella* spp. (Supplementary Table 1).

Phage susceptibility was determined with the spot test with double agar overlay method [18]. Briefly, 100 μl of a 0.5 McFarland bacterial suspension was mixed in a brain heart infusion (BHI) agarose matrix (0.6%), which was then distributed to solidify on a standard BHI agar plate. Then, 10 μl of each phage-suspension was spotted on the plate and incubated overnight. The day after, lysis zones were quantified [18]. Specifically, strains showing confluent lysis (complete clearing: ++++), semi-confluent lysis (clearing throughout, but with faint hazy background: +++), opaque lysis (turbidity throughout the cleared zone: ++), and *taches vierges* (individual clear or opaque plaques: +) were defined as susceptible to the phage compounds tested. Strains showing no activity (no clearing: *R*) were defined as resistant. For all strains (n = 50) susceptibility tests were performed in duplicate and on distinct days.

## RESULTS AND DISCUSSION

**Supplementary Figure 1.**
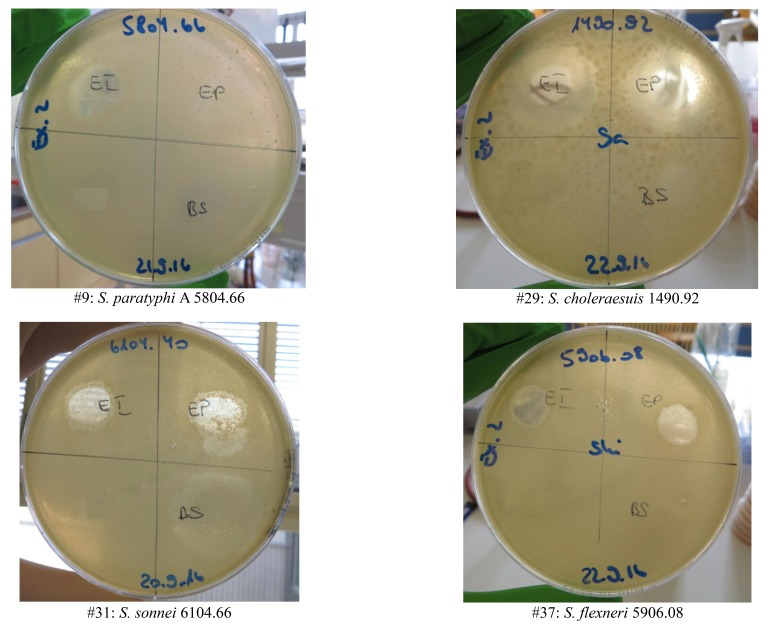
Examples of bacteriophage susceptibility results (see Supp. Table 1) for 2 *Salmonella* and 2 *Shigella* spp. strains. EI, Eliava *INTESTI Bacteriophage* cocktail; EP, Eliava *PYO Bacteriophage* (Eliava) cocktail; BS, Biochimpharm *Septaphage Bacteriophage* cocktail.

As shown in Table 1, the overall susceptibility of *Salmonella* spp. to *INTESTI* and *Septaphage* was 86.7% (of which 23/30 were +++ or ++++) and 76.7% (none of which were +++ or ++++), respectively (examples in Supplementary Figure 1). With regard to *Shigella* spp., the overall susceptibility to *INTESTI* and *Septaphage* was 95% (of which 9/20 were +++ or ++++) and 55% (of which 3/20 were +++ or ++++), respectively. This data is promising, but we should note that the spot test can lead to an overestimation of the susceptibility as a consequence of the *lysis-from-without* phenomenon [19].

**Table 1.** Summary of the susceptibility of the *Salmonella* and *Shigella* spp. strains to the 3 commercial bacteriophage cocktails

Phage Cocktails	Strain groups	Results of the spot test (%) ^a^				
		R	+	++	+++	++++
*PYO Bacteriophage* (Eliava)	Overall strains (n = 50)	56.0	4.0	24.0	12.0	4.0
	*Salmonella* spp. (n = 30)	90.0	3.3	0.0	3.3	3.3
	*Shigella* spp. (n = 20)	5.0	5.0	55.0	30.0	5.0
*INTESTI Bacteriophage* (Eliava)	Overall strains (n = 50)	10.0	6.0	20.0	36.0	28.0
	*Salmonella* spp. (n = 30)	13.3	3.3	6.7	33.3	43.3
	*Shigella* spp. (n = 20)	5.0	10.0	40.0	40.0	5.0
*Septaphage* (Biochimpharm)	Overall strains (n = 50)	32.0	42.0	20.0	0.0	6.0
	*Salmonella* spp. (n = 30)	23.3	53.3	23.3	0.0	0.0
	*Shigella* spp. (n = 20)	45.0	25.0	15.0	0.0	15.0

^a^ Strains were defined as susceptible to the bacteriophages when confluent lysis (ie, complete clearing: ++++), semi-confluent lysis (ie, clearing throughout but with faint hazy background: +++), opaque lysis (ie, turbidity throughout the cleared zone: ++), *taches vierges* (ie, a few individual plaques: +) were recorded. Strains showing no activity (ie, no clearing “R”) were defined as resistant.

We did not expect any activity for *PYO* against our strains because, according to the manufacturer, this preparation should not contain lytic phages against *Salmonella* spp. and *Shigella* spp. However, we were surprised to note that this cocktail was active against 10% (of which 2/30 were +++ or ++++) of *Salmonella* spp. and, more importantly, against 95% (of which 7/20 were +++ or ++++) of *Shigella* spp. This could be explained by the presence of bacteriophages unable to selectively differentiate *Salmonella* and *Shigella* spp. from *E. coli* (all 3 being phylogenetically closely related bacterial species, especially the latter 2 [20]) that might share several common phage targets [21]. Moreover, taking into account the *lysis-from-without* phenomenon where a high multiplicity of infection can lead to bacterial death without infection, we are aware that by exclusively using the spot test, our susceptibility results might be slightly overestimated [19].

In conclusion, we showed the distinct spectrum and lytic activity of commercial bacteriophage cocktails targeting *Salmonella* and *Shigella* species. In particular, *Septaphage* proved to be active, though overall weakly, against 68% of the tested strains, whereas *INTESTI* exhibited a strong response against 90% of our isolates. Therefore, our results seem promising, especially for the latter biopreparation against *Salmonella enterica* infections. Nevertheless, such speculation should be supported by further animal studies together with human clinical trials in order to confirm efficacy and safety of cocktails. We also emphasize the importance of large *in vitro* screening analyses aimed to assess the activity of such biopreparations against contemporary multidrug-resistant strains emerging worldwide [2, 22, 23]. The sum of these steps, if successful, could lead to the maturation—also in Western countries—of an alternative approach for the treatment of bacillary dysenteries and salmonellosis.

## Appendix

**Supplementary Table 1.** Characteristics of the 30 *Salmonella* and 20 *Shigella* spp. strains and susceptibility to 3 commercial bacteriophage cocktails

No.	ID strain	Species	Source	Detection Month/Year	Susceptibility according to CLSI						Bacteriophage Susceptibility ^a^		
					AMP	CRO	SXT	CHL	NAL	CIP	*INTESTI*	*Septaphage*	*PYO*
1	6301.21	*S. enteritidis*	Stool	08/16	S	S	S	S	S	S	++++	+	R
2	6301.22	*S. enteritidis*	Stool	08/16	S	S	S	S	S	S	R	+	R
3	6301.23	*S. enterica subsp. enterica 4,12:i*	Stool	08/16	S	S	S	S	S	S	R	+	R
4	6212.52	*S. enteritidis*	Stool	08/16	S	S	S	S	S	S	R	+	R
5	6212.46	*S. enterica subsp. enterica 4,12:i*	Stool	08/16	R	S	S	S	S	S	R	+	R
6	6212.47	*S. enteritidis*	Stool	08/16	S	S	S	S	S	S	+++	+	R
7	6211.59	*S. enterica subsp. enterica 6,7:y:-*	Stool	08/16	S	S	S	S	S	S	++++	++	+++
8	6211.25	*S. enteritidis*	Stool	08/16	S	S	S	S	S	S	+++	+	R
9	5804.66	*S. paratyphi A*	Blood culture	04/15	S	S	S	S	R	I	+++	+	R
10	6102.20	*S. typhimurium*	Urine	01/16	S	S	S	S	S	S	+++	R	R
11	6103.32	*S. typhimurium*	Stool	02/16	S	S	S	S	S	S	++++	+	R
12	6107.71	*S. typhimurium*	Stool	03/16	S	S	S	S	S	S	++++	++	R
13	6007.27	*S. panama*	Stool	11/15	S	S	S	S	S	S	++++	+	R
14	5804.47	*S. paratyphi B*	Stool	04/15	S	S	S	S	S	S	+++	+	R
15	5602.57	*S. typhimurium*	Blood culture	09/14	S	S	S	S	S	S	++++	++	R
16	5905.07	*S. enteritidis*	Stool	08/15	S	S	S	S	S	S	+++	+	R
17	5905.08	*S. enteritidis*	Stool	08/15	S	S	S	S	S	S	++++	++	R
18	5602.08	*S. enteritidis*	Stool	09/14	S	S	S	S	S	S	+++	++	R
19	5512.03	*S. enteritidis*	Blood culture	08/14	S	S	S	S	S	S	++++	+	R
20	5603.72	*S. enteritidis*	Blood culture	09/14	S	S	S	S	S	S	+++	++	R
21	4608.23	*S. paratyphi A*	Stool	12/10	S	S	S	S	R	S	++++	R	R
22	4504.56	*S. paratyphi A*	Blood culture	06/10	S	S	S	S	R	I	++++	R	R
23	6104.03	*S. paratyphi B*	Blood culture	02/16	S	S	S	S	S	S	+++	R	R
24	6201.74	*S. paratyphi B*	Stool	05/16	S	S	S	S	S	S	++	+	R
25	5902.41	*S. typhimurium*	Stool	07/15	S	S	S	S	S	S	++++	R	R
26	5910.36	*S. typhimurium*	Stool	09/15	S	S	S	S	S	S	++++	R	R
27	4108.64	*S. oranienburg*	Stool	03/09	S	S	S	S	S	S	+	R	R
28	4310.33	*S. oranienburg*	Stool	12/09	S	S	S	S	S	S	+++	+	+
29	1490.92	*S. choleraesuis*	na	na	-	-	-	-	-	-	++++	++	++++
30	6302.34	*S. enteritidis*	Stool	9/16	S	S	S	S	S	S	++	+	R
31	6101.40	*S. sonnei*	Stool	01/16	S	S	R	S	S	S	+++	+	+++
32	6105.15	*S. sonnei*	Stool	03/16	S	S	R	S	S	S	+++	+	+++
33	6108.73	*S. sonnei*	Stool	04/16	-	-	-	-	-	-	+++	++++	+++
34	6110.62	*S. sonnei*	Stool	04/16	R	S	R	S	S	S	++	+	+++
35	6003.54	*S. flexneri*	Stool	10/15	-	-	-	-	-	-	++++	R	+++
36	6004.50	*S. flexneri*	Stool	11/15	S	S	R	S	R	S	++	R	++
37	5906.08	*S. flexneri*	Stool	08/15	S	S	S	S	S	S	++	R	++
38	5509.52	*S. flexneri*	Stool	08/14	R	S	R	R	S	S	R	R	R
39	6306.26	*S. sonnei*	Stool	10/16	S	S	R	S	S	S	+++	++++	++
40	5703.48	*S. sonnei*	Stool	11/14	S	S	R	S	R	R	+	+	+
41	5611.08	*S. sonnei*	Stool	11/14	-	-	-	-	-	-	+++	++++	+++
42	5605.11	*S. sonnei*	Stool	10/14	S	S	R	S	S	S	++	++	++
43	5402.22	*S. sonnei*	Stool	03/14	S	S	R	S	R	R	++	++	++
44	5312.31	*S. sonnei*	Stool	02/14	R	S	S	S	S	S	++	++	++
45	5203.63	*S. sonnei*	Stool	05/13	S	S	R	S	S	S	++	+	++
46	6209.65	*S. flexneri*	Stool	08/16	-	-	-	-	-	-	+++	R	++
47	4907.58	*S. flexneri*	Stool	02/12	S	S	R	R	R	R	+++	R	++++
48	4706.22	*S. flexneri*	Stool	04/11	S	S	R	S	R	S	+	R	++
49	4611.14	*S. flexneri*	Stool	01/11	S	S	R	S	S	S	++	R	++
50	4512.64	*S. flexneri*	Stool	09/10	R	S	S	R	S	S	+++	R	++

**Note.** AMP, ampicillin; CRO, ceftriaxone; SXT, cotrimoxazole; CHL, chloramphenicol; NAL, nalidixic acid; CIP, ciprofloxacin; R, resistant; I, intermediate; S, susceptible; na, not available; -, not tested.

^a^ Strains were defined as susceptible to the bacteriophages when confluent lysis (ie, complete clearing: ++++), semi-confluent lysis (ie, clearing throughout but with faint hazy background: +++), opaque lysis (ie, turbidity throughout the cleared zone: ++), *taches vierges* (ie, a few individual plaques: +) were recorded. Strains showing no activity (ie, no clearing “R”) were defined as resistant.
